# Empagliflozin Enhances Autophagy, Mitochondrial Biogenesis, and Antioxidant Defense and Ameliorates Renal Ischemia/Reperfusion in Nondiabetic Rats

**DOI:** 10.1155/2022/1197061

**Published:** 2022-01-28

**Authors:** Moein Ala, Mohammad Reza Fallahpour Khoshdel, Ahmad Reza Dehpour

**Affiliations:** ^1^Experimental Medicine Research Center, Tehran University of Medical Sciences (TUMS), Tehran, Iran; ^2^Department of Pharmacology, School of Medicine, Tehran University of Medical Sciences (TUMS), Tehran, Iran

## Abstract

**Background:**

Recent meta-analyses have shown that sodium-glucose cotransporter 2 (SGLT-2) inhibitors alleviate chronic kidney disease and acute kidney injury in diabetic patients. In this study, we aimed to investigate the effect of empagliflozin on renal ischemia/reperfusion (I/R) in nondiabetic rats and find the possible mechanisms. *Experimental Approach*. Eighteen male Wistar rats were randomly divided into three groups, including healthy control, ischemic control, and empagliflozin-treated group. Thirty minutes of bilateral renal ischemia was induced by clamping the renal hilum. Forty-eight hours after reopening the clamps, rats' blood samples and tissue specimens were collected. Empagliflozin 10 mg/kg was administered by gavage, 2 hours before ischemia and 24 hours after the first dose.

**Results:**

I/R injury led to a significant rise in serum creatinine and blood urea nitrogen which was significantly decreased after treatment with empagliflozin. Empagliflozin also alleviated tubulointerstitial and glomerular damage and significantly decreased tissue histology scores. Empagliflozin decreased the increased levels of malondialdehyde, interleukin 1*β*, and tumor necrosis factor *α*. SGLT2 inhibition increased the decreased expression of nuclear factor erythroid 2-related factor 2 and PPARG coactivator 1 alpha that conduct antioxidant defense and mitochondrial biogenesis, respectively. Furthermore, empagliflozin markedly increased LC3-II/LC3-I and bcl2/bax ratios, showing its beneficial effect on activation of autophagy and inhibition of apoptosis. Despite its effects on diabetic nephropathy, empagliflozin did not activate the Sestrin2/AMP-activated protein kinase pathway in this study.

**Conclusion:**

Empagliflozin improved renal I/R injury in nondiabetic rats in this study by promoting autophagy and mitochondrial biogenesis and attenuation of oxidative stress, inflammation, and apoptosis.

## 1. Introduction

SGLT-2 inhibitors are a new generation of antidiabetic medications. SGLT-2 inhibition hinders the reabsorption of Na^+^ and glucose in the proximal convoluted tubule in the kidney [1]. Previously, it has been uncovered that the use of SGLT-2 inhibitors is associated with markedly better cardiovascular and renal outcomes among diabetic patients [2]. Six weeks of treatment with dapagliflozin, an SGLT-2 inhibitor, reduced albuminuria by 43.9% (95% confidence interval (CI) (30.3%-54.8%)) and urinary kidney injury molecule-1 in diabetic patients [3]. A meta-analysis of 40 randomized clinical trials showed that SGLT-2 inhibitors can reduce the urine albumin/creatinine ratio (-23.4, 95% CI (-44.6 to -2.2) and decrease the need for renal replacement therapy (0.65, 95% CI (0.54–0.79)), renal death (0.57, 95% CI (0.49–0.65)), and progression of albuminuria (0.69, 95% CI (0.66–0.73)) among diabetic patients [4]. Interestingly, a meta-analysis of 112 randomized clinical trials and 4 observational studies with 5 cohorts consisting of 180656 diabetic patients revealed that SGLT2 inhibitors decreased the odds of acute kidney injury (AKI) by 36% (odds ratio (OR) 0.64, 95% CI (0.53-0.78)) among those patients [5].

AKI is a common medical condition particularly among hospitalized or critically ill patients [6]. AKI is defined as the rapid decline in the excretory function of the kidneys, characterized by deposition of end products of nitrogen metabolism such as urea and creatinine or diminished urine output [7]. Renal ischemia is a major type of AKI that is associated with high mortality and morbidity rate [8]. Reoxygenation of the ischemic kidney leads to mitochondrial dysfunction, generation of a massive amount of reactive oxygen species (ROS), inflammation, decompensation of repair mechanisms, and cell death [8]. It has been observed that empagliflozin can alleviate inflammation and mitigate AKI after myocardial infarction in diabetic rats [9]. Nespoux et al. indicated that genetic deletion of SGLT-2 cannot protect against renal I/R injury in nondiabetic mice [10]. However, other studies revealed that downregulation of SGLT-2 can improve renal I/R in nondiabetic rats [11]. It has been shown that the Sestrin2/AMP-activated protein kinase (AMPK) pathway is critically involved in the protective effects of SGLT-2 inhibitors on metabolism, fibrosis, and organ damage in mice [12]. In particular, it was shown that activation of AMPK by SGLT-2 inhibition is a major protective mechanism in diabetic nephropathy [13]. Activation of the Sestrin2/AMPK pathway modulates inflammation, autophagy, mitochondrial biogenesis, protein synthesis, oxidative stress, and endoplasmic reticulum stress [14]. Here, we aimed to investigate the effect of empagliflozin, an SGLT-2 inhibitor, on renal I/R injury in nondiabetic rats and find the possible underlying mechanisms. We measured the effects of empagliflozin on autophagy, apoptosis, and the expression of major signaling molecules.

## 2. Material and Methods

### 2.1. Animals and Grouping

Eighteen male Wistar rats weighing between 200 and 250 g were purchased from the Department of Pharmacology, Tehran University of Medical Sciences (TUMS). Rats were treated humanely and kept in a temperature-controlled room (25 ± 2°C), with a 12 : 12-hour light and dark cycle. They had free access to standard food and water. After acclimatization to their environment, they were randomly divided into three groups, including a healthy control group, an ischemic control group, and an empagliflozin-treated group. The first group did not undergo any procedure until blood sampling and tissue extraction. I/R injury was induced in the ischemic control group and empagliflozin-treated group. Rats were anesthetized with ketamine (87 mg/kg) and xylazine (13 mg/kg) before surgery, blood sampling, and tissue biopsy. After tissue extraction, rats were sacrificed with a CO_2_ chamber. Rats were treated according to the *Guide for the Care and Use of Laboratory Animals* (8th edition, National Academies Press) and institutional guidelines for animal care and use (Department of Pharmacology, School of Medicine, Tehran University of Medical Sciences, Tehran, Iran).

### 2.2. I/R Injury and Treatment Schedule

**Figure 1:**
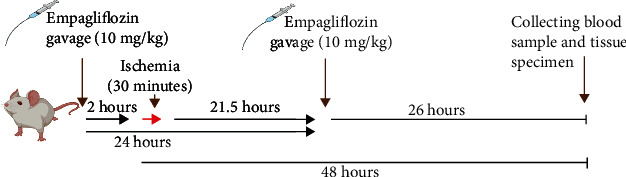
Study timeline.

Rats were anesthetized and their abdomens were shaved. A bilateral flank incision was utilized to provide access to both kidneys. I/R injury was induced by ligating the hilum of the kidneys with bulldog clamps. Ischemia lasted for 30 minutes; then, the clamps were reopened, and abdominal incisions were sutured. Rats were followed until awakening, and 48 hours after ischemia, they were anesthetized again; their blood samples were taken, and their kidneys were harvested for histopathology and molecular measurements. Treated rats received empagliflozin by gavage. The most effective dose of empagliflozin (10 mg/kg) that showed protective effects on the kidney of diabetic rats was used in this study [9]. Rats received empagliflozin (Abidi, Iran) 2 hours before induction of I/R injury. This dose of the drug was repeated 24 hours after the first dose; i.e., empagliflozin was administered twice, 2 hours before the initiation of ischemia and 22 hours after the initiation of ischemia (Figure 1).

### 2.3. Renal Function Test and Histopathology

After anesthesia, blood samples were taken from renal arteries and centrifuged at a speed of 3000 rounds per minute (rpm) for 5 minutes. Serum samples were collected and kept at -20°C until assay. A fully automated Hitachi analyzer was used for measuring the serum levels of blood urea nitrogen (BUN) and creatinine. Due to histological measurements, kidney specimens were fixed in formaldehyde 4% after biopsy. After tissue processing, specimens were stained with hematoxylin and eosin (H&E). An expert pathologist blinded to our experiment interpreted the histological views of samples. Based on the endothelial, glomerular, tubular, and interstitial (EGTI) histology scoring system for renal ischemia/reperfusion injury [15] (Table 1), each specimen obtained a score for tubular, glomerular, endothelial, and tubulointerstitial characteristics of the tissue. A sum of these scores was determined for each specimen and used for comparing the groups.

### 2.4. ELISA

Tissue specimens were preserved at -80°C before assay. After homogenization, the tissue levels of inflammatory cytokines such as tumor necrosis factor *α* (TNF-*α*) and interleukin 1*β* (IL1*β*) were measured utilizing a Rat TNF-*α* DuoSet® ELISA Development kit (DY510) and a Rat IL1*β*/IL1F2 DuoSet® ELISA Development kit (DY501). Kits were utilized following the manufacturer's instruction. After plate preparation, 100 *μ*L of specimens or standards was added to the reagent diluent and incubated for 2 hours at 25°C. Wells were aspirated and washed with wash buffer. Thereafter, 100 *μ*L of diluted detection antibody was added to wells. Then, the plate was washed, 100 *μ*L of working dilution of streptavidin-HRP B was added to each well, and the plate was covered to prevent direct light exposure and incubated for 20 minutes at 25°C. Again, the plate was washed, 100 *μ*L of substrate solution was added to each well, and the plate was covered to prevent direct light exposure and incubated for 20 minutes at room temperature. Eventually, 50 *μ*L of stop solution was added to each well, and the plate was gently tapped to ensure mixing properly. The optical density of wells was determined with a microplate reader set to 540 nm. The standard curve was drawn, and the tissue levels of TNF-*α* and IL1*β* were calculated, subsequently.

### 2.5. Malondialdehyde (MDA) Assay

Homogenates of tissue specimens were used for measuring tissue levels of MDA, as a marker of lipid peroxidation and oxidative stress [16]. We purchased and utilized a Biocore (ZellBio) MDA assay kit for measuring the tissue levels of MDA. The assay was performed based the manufacturer's instruction as follows. In the first step, 100 *μ*L of standards or specimens was added to each test tube. Thereafter, 100 *μ*L of diluted BCD-R4 was added to each test tube. Then, 200 *μ*L chromogenic solution was added. In the next step, the mixture was heated for one hour at a boiling water bath, cooled in the ice bath, and centrifuged for 10 minutes at 10000 rpm. Eventually, 200 *μ*L of pink color supernatant was transferred into a microplate, and the absorbance was read at 535 nm. The standard curve was drawn based on the results, and MDA levels were determined subsequently.

### 2.6. Western Blotting

Tissue specimens were snap-frozen and preserved at -80°C until assay. The lysis buffer, consisting of Tris-HCL (500 *μ*L, pH = 8), NaCl (0.08 g), sodium deoxycholate (0.025 g), SDS (0.01 g), EDTA (0.003 g), protease inhibitor cocktail (1 tablet), and triton (NP40 (1%)) (10 *μ*L), was mixed with the tissue homogenate. The mixture was centrifuged (Eppendorf 5415 R) for 10 minutes at 12000 rpm and 4°C. The supernatants were collected and preserved at -20°C. A Bradford protein assay utilizing Bradford solution (Coomassie blue G250 (5 mg), phosphoric acid (5 mg), ethanol 95% (2.5 mg), and distilled water (up to 50 mL)) was used to measure the concentration of total protein in each specimen. Samples reached an equal concentration of total protein before adding them to wells. After completing the running phase, protein bands were transferred from the SDS-PAGE onto the PVDF membrane. Thereafter, the PVDF membrane was blocked with blocking solution including 2% nonfat powdered milk dissolved in TBS-T buffer (Tris-HCL (20 mL), Tween 20 0.1 *v*/*v* (1 mL), sodium chloride (8 g), and distilled water (100 mL)). The blocking phase lasted 75 minutes. Thereafter, the PVDF membrane was incubated with primary antibodies (*β*-actin (sc-47778, 1 : 300), AMPK (sc-74461, 1 : 300), p-AMPK (sc-33524, 1 : 300), PPARG coactivator 1 alpha (PGC1-*α*) (ab54481, 1 : 300), mitochondrial transcription factor A (mTFAM) (sc-166965, 1 : 300), nuclear factor erythroid 2-related factor 2 (Nrf2) (sc-365949, 1 : 300), Sestrin2 (sc-101249, 1 : 300), bax (sc-7480, 1 : 300), bcl2 (sc-492, 1 : 300), and LC3B (LC3B antibody #2775, 1 : 300)) for 16 hours. Then, the membrane was washed with TBS-T buffer and incubated with a secondary antibody (sc-2357, 1 : 1000) for 75 minutes at room temperature. An open-source version of ImageJ software was used to determine the optical density of protein bands.

### 2.7. Statistics

Data were analyzed by GraphPad Prism version 6.07. Results were compared using one-way analysis of variance (ANOVAs) followed by post hoc Tukey's tests. Charts are displayed as mean ± SEM (standard error of the mean). Differences were deemed statistically significant when *p* value < 0.05.

## 3. Results

### 3.1. SGLT-2 Inhibition by Empagliflozin Ameliorated Renal Dysfunction after Renal I/R Injury

**Figure 2:**
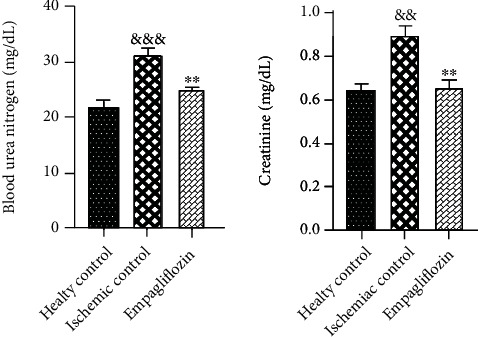
The effect of SGLT-2 inhibition on renal function after I/R injury. I/R injury was associated with a significant rise in the serum levels of creatinine (^&&^*p* < 0.01) and BUN (^&&&^*p* < 0.001). Treatment with empagliflozin markedly reduced the serum levels of creatinine (^∗∗^*p* < 0.01) and BUN (^∗∗^*p* < 0.01).

Blood samples were taken from renal arteries, 48 hours after I/R injury. Serum levels of creatinine and BUN were measured to evaluate renal function. I/R injury resulted in renal dysfunction and significantly increased the serum levels of creatinine (^&&^*p* < 0.01) and BUN (^&&&^*p* < 0.001), compared with the healthy control group. Treatment with empagliflozin markedly decreased the increased levels of creatinine (^∗∗^*p* < 0.01) and BUN (^∗∗^*p* < 0.01), compared with the ischemic control group (Figure 2).

### 3.2. SGLT-2 Inhibition by Empagliflozin Ameliorated Renal Dysfunction after Renal I/R Injury

**Figure 3:**
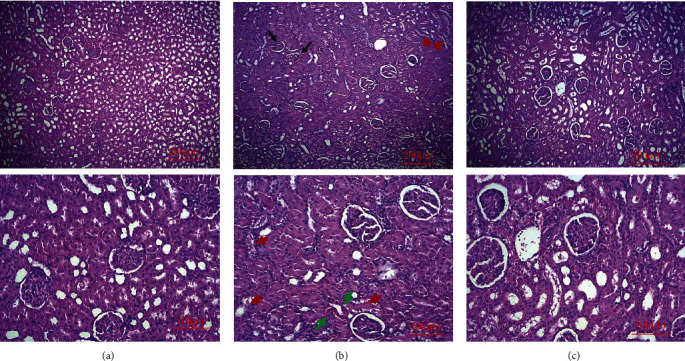
The effect of I/R injury and empagliflozin on kidney microstructure. I/R injury (b) deranged the microarchitecture of the kidney and led to tubular inflammation and cast formation and tubular necrosis (red arrows) and thickening of the Bowman capsule, retraction of glomerular tuft (black arrows), glomerular fibrosis, and interstitial inflammation and hemorrhage (green arrow), compared with the healthy control group (a). Treatment with empagliflozin (c) notably improved tissue injury.

**Figure 4:**
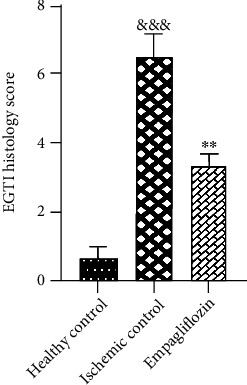
The protective effect of I/R injury and empagliflozin on kidney tissue based on the EGTI histology score. I/R injury significantly (^&&&^*p* < 0.001) damaged kidney tissue based on the EGTI histology score, and treatment with empagliflozin markedly (^∗∗^*p* < 0.01) alleviated tissue damage.

Tissue specimens were fixed in formaldehyde, and after tissue processing, they were stained with H&E. Based on the EGTI histology scoring system for renal I/R injury, each sample received a histology score. Signs of tissue damage such as tubular inflammation and cast formation and tubular necrosis (red arrows, Figure 3) and thickening of the Bowman capsule, retraction of glomerular tuft (black arrows, Figure 3), glomerular fibrosis, and interstitial inflammation and hemorrhage (green arrows, Figure 3) were abundantly found in the ischemic control group and partly restored in the empagliflozin-treated group (Figure 3). The EGTI histology score (Table 1) showed that I/R injury significantly distorted kidney microarchitecture (^&&&^*p* < 0.001) that was significantly mitigated after treatment with empagliflozin (^∗∗^*p* < 0.01) (Figure 4).

### 3.3. SGLT-2 Inhibition by Empagliflozin Decreased Renal MDA Levels, ILl*β*, and TNF-*α* after Renal I/R Injury

**Figure 5:**
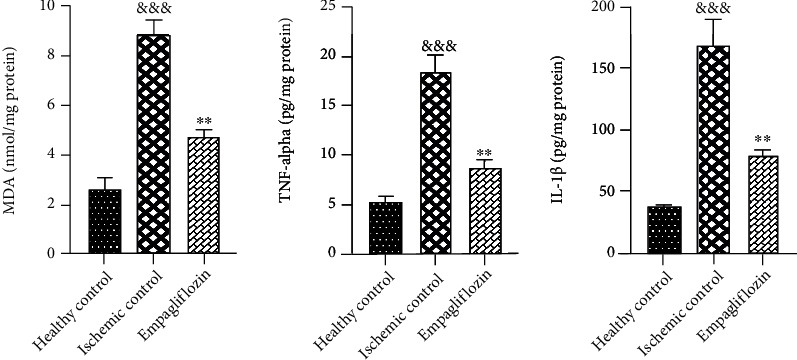
The effect of empagliflozin on inflammatory cytokines and oxidative stress. I/R injury was associated with a significant increase in the tissue levels of MDA (^&&&^*p* < 0.001), ILl*β* (^&&&^*p* < 0.001), and TNF-*α* (^&&&^*p* < 0.001). Empagliflozin significantly decreased the tissue levels of MDA (^∗∗^*p* < 0.01), ILl*β* (^∗∗^*p* < 0.01), and TNF-*α* (^∗∗^*p* < 0.01).

Snap-frozen tissue biopsies were kept at -80°C and used for measuring the tissue levels of inflammatory mediators. Tissue levels of MDA, ILl*β*, and TNF-*α* were measured 48 hours after I/R injury. MDA was measured by the colorimetric method, and ILl*β* and TNF-*α* were measured by the ELISA method. I/R injury led to a significant increase in the tissue levels of MDA (^&&&^*p* < 0.001), ILl*β* (^&&&^*p* < 0.001), and TNF-*α* (^&&&^*p* < 0.001), compared with the healthy control group. Treatment with empagliflozin significantly decreased the tissue levels of MDA (^∗∗^*p* < 0.01), ILl*β* (^∗∗^*p* < 0.01), and TNF-*α* (^∗∗^*p* < 0.01), compared with the ischemic control group (Figure 5).

### 3.4. SGLT-2 Inhibition by Empagliflozin Upregulated Nrf2 but Did Not Change the Expression of Sestrin2 and AMPK in Renal I/R Injury

**Figure 6:**
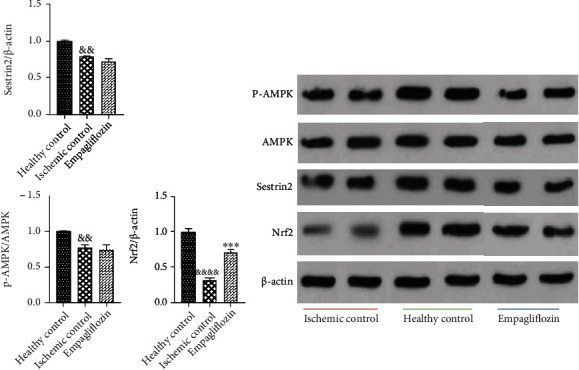
The effect of empagliflozin on the expression levels of Sestrin2, AMPK, and Nrf2. I/R injury significantly reduced the expression of Sestrin2 (^&&^*p* < 0.01), AMPK (^&&^*p* < 0.01), and Nrf2 (^&&&&^*p* < 0.0001). Treatment with empagliflozin did not significantly change the expression of Sestrin2 and AMPK, while it notably upregulated Nrf2 (^∗∗∗^*p* < 0.001).

Previously, it has been uncovered that SGLT-2 inhibitors partly act through the Sestrin2/AMPK pathway to modulate oxidative stress and energy metabolism and exert their renoprotective effects on diabetic nephropathy [2]. Herein, we decided to measure if the same pathway is involved in the protective effect of empagliflozin on renal I/R injury in nondiabetic rats or not. Tissue expression of Sestrin2, AMPK, and Nrf2 was measured by western blotting, and their relative expression was determined according to the expression of *β*-actin. I/R injury significantly downregulated Sestrin2 (^&&^*p* < 0.01) and AMPK (^&&^*p* < 0.01), compared with the healthy control group. Empagliflozin could not significantly change the expression levels of Sestrin2 and AMPK, compared with the ischemic control group. In addition, we aimed to find out how empagliflozin can protect against oxidative stress in renal I/R injury in nondiabetic rats. Accordingly, we measured the expression levels of Nrf2 which is a master transcription factor for several antioxidant genes. I/R injury markedly (^&&&&^*p* < 0.0001) decreased Nrf2 expression, compared with the healthy control group. Treatment with empagliflozin significantly (^∗∗∗^*p* < 0.001) increased the expression of Nrf2, compared with the ischemic control group (Figure 6).

### 3.5. SGLT-2 Inhibition by Empagliflozin Enhanced PGC-1*α* Expression, Increased Autophagy, and Prevented Apoptosis

To evaluate the effect of I/R injury and empagliflozin on mitochondrial biogenesis, we measured the expression of PGC-1*α* and TFAM in the tissue biopsies by western blotting. I/R injury significantly downregulated the PGC-1*α* (^&&&^*p* < 0.001) and TFAM (^&^*p* < 0.05), compared with the healthy control group. Treatment with empagliflozin significantly upregulated PGC-1*α* (^∗∗^*p* < 0.01), while it did not significantly change the expression of TFAM, compared with the ischemic control group.

**Figure 7:**
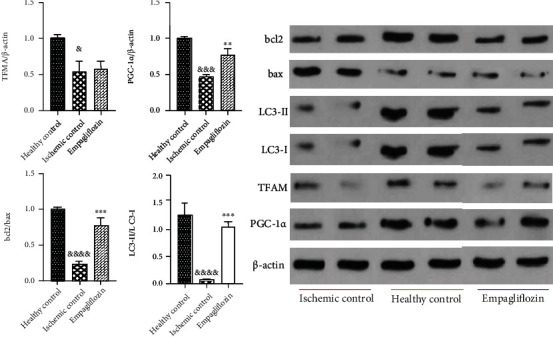
The effect of empagliflozin on mitochondrial biogenesis, autophagy, and apoptosis in renal I/R injury. I/R injury downregulated PGC-1*α* (^&&&^*p* < 0.001) and TFAM (^&^*p* < 0.05). Empagliflozin upregulated PGC-1*α* (^∗∗^*p* < 0.01), while it did not alter the expression level of TFAM. I/R injury was associated with a significant decrease in the bcl2/bax ratio (^&&&&^*p* < 0.0001) and LC3-II/LC3-I ratio (^&&&&^*p* < 0.0001). Treatment with empagliflozin markedly increased the bcl2/bax ratio (^∗∗∗^*p* < 0.001) and LC3-II/LC3-I ratio (^∗∗∗^*p* < 0.001).

Due to uncovering the effect of I/R injury and empagliflozin on autophagy and apoptosis, we measured the bcl2/bax ratio and LC3-II/LC3-I ratio in tissue biopsies. I/R injury led to a significant decrease in the bcl2/bax ratio (^&&&&^*p* < 0.0001) and LC3-II/LC3-I ratio (^&&&&^*p* < 0.0001), compared with the healthy control group. In contrast, empagliflozin markedly increased both the bcl2/bax ratio (^∗∗∗^*p* < 0.001) and LC3-II/LC3-I ratio (^∗∗∗^*p* < 0.001), compared with the ischemic control group (Figure 7).

## 4. Discussion

Our findings showed that empagliflozin ameliorated renal dysfunction and renal histological damage after I/R injury. Empagliflozin partly promoted mitochondrial biogenesis through PGC-1*α* and increased Nrf2 expression. In addition, empagliflozin decreased lipid peroxidation and inflammatory cytokine levels. These findings show that empagliflozin could partly prevent the production of ROS or augment the antioxidant system.

In addition, empagliflozin markedly promoted autophagy and decreased apoptosis which shows the successful activation of compensatory mechanisms. However, our results revealed that the Sestrin2/AMPK pathway was not involved in the protective effects of empagliflozin on renal I/R injury in nondiabetic rats.

Nrf2 is a transcription factor for several antioxidant genes and the main regulator of antioxidant response [17, 18]. Nrf2 upregulation vigorously attenuates oxidative stress, protects against apoptosis, and prevents renal tubular cell injury in renal I/R [17, 18]. Consistently, Liu et al. revealed that Nrf2 knockout leads to more severe inflammatory response, structural and functional impairment in the mouse model of renal I/R, and cisplatin-induced nephrotoxicity [19].

Mitochondrial dysfunction is a major driver for kidney diseases, and pharmacological repair of mitochondrial function can ameliorate kidney injury [20, 21]. Mitochondrial dysfunction and subsequent oxidative burst are the main propellants for inflammation and tissue damage in renal I/R [22, 23]. Attenuation of oxidative stress and amelioration of mitochondrial dysfunction can mitigate the initial damage and improve the outcome of renal I/R [22, 23]. Uncontrolled release of ROS leads to DNA fragmentation, production of numerous inflammatory mediators such as TNF-*α*, IL1*β*, and IL6, and activation of proapoptotic factors and results in cell death [23].

PGC-1*α* is a transcription coactivator for several genes involved in mitochondrial biogenesis and a key regulator of energy metabolism [24, 25]. Inactivation of PGC-1*α* decreases the expression of mitochondrial genes, augments inflammation in AKI, and increases renal tubular cell loss [24]. PGC-1*α* activation also enhances autophagy and ketogenesis which can greatly alleviate kidney injury [26]. Therapeutic approaches that increase the expression of PGC-1*α* showed protective effects on AKI [27]. Previously, it was observed that empagliflozin upregulates Sestrin2/AMPK to improve mitochondrial dysfunction in rats with the metabolic disorders [12]. In this study, empagliflozin upregulated Nrf2 and PGC-1*α* and decreased lipid peroxidation, but the Sestrin2/AMPK pathway was not involved in these effects.

We also measured the expression of TFAM which maintains the integrity of mitochondrial DNA [28]. Renal I/R injury downregulates TFAM expression, damages mitochondrial DNA, and reduces mitochondrial energy metabolism [28]. Empagliflozin could not significantly change the expression of TFAM in this study.

Autophagy is a compensatory mechanism for the removal of damaged and dysfunctional organelles and misfolded proteins [29]. Pharmacological inhibition or genetic attenuation of autophagy exacerbated lipopolysaccharide-induced AKI in mice [29, 30]. Conversely, augmentation of autophagy with rapamycin markedly alleviated cisplatin-induced AKI in mice [31]. Likewise, Sunahara et al. showed that autophagy is downregulated in sepsis-mediated AKI, and acceleration of autophagy by rapamycin improves AKI in the mouse model of sepsis [32]. Increased autophagic flux reduces inflammatory cytokine production and increases the expression of anti-inflammatory cytokines and confines structural and functional impairments in AKI [33, 34]. Autophagy provides an opportunity for self-repair, and impaired autophagy results in increased apoptotic cell death [30, 31]. Previously, it was found that empagliflozin activates autophagy in the kidney of diabetic rats [35]. In this study, empagliflozin pronouncedly enhanced autophagy and prevented apoptosis in nondiabetic rats.

## 5. Conclusion

**Figure 8:**
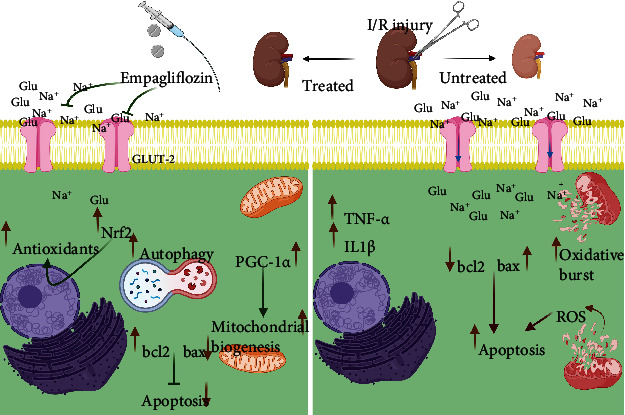
The protective effects of empagliflozin on renal I/R injury in nondiabetic rats.

Based on our findings, empagliflozin ameliorated renal I/R injury in nondiabetic rats. Alleviation of oxidative stress, inflammation, and apoptosis, improvement of mitochondrial biogenesis, and a marked increase in autophagy were shown to be associated with the protective effect of empagliflozin on renal I/R injury (Figure 8).

## Figures and Tables

**Table 1:** EGTI histology scoring system for renal ischemia/reperfusion injury defined by Chavez et al. [15].

Tissue type	Damage	Score
Tubular	No damage	0
	Loss of brush border (BB) in less than 25% of tubular cells. Integrity of the basal membrane	1
	Loss of BB in more than 25% of tubular cells, thickened basal membrane	2
	(Plus) inflammation, cast formation, necrosis up to 60% of tubular cells	3
	(Plus) necrosis in more than 60% of tubular cells	4

Endothelial	No damage	0
	Endothelial swelling	1
	Endothelial disruption	2
	Endothelial loss	3

Glomerular	No damage	0
	Thickening of the Bowman capsule	1
	Retraction of glomerular tuft	2
	Glomerular fibrosis	3

Tubulointerstitial	No damage	0
	Inflammation, hemorrhage in less than 25% of tissue	1
	Inflammation, hemorrhage in less than 25% of tissue plus necrosis in less than 25% of tissue	2
	Necrosis up to 60%	3
	Necrosis more than 60%	4

## Data Availability

Data analyzed for this article are available from the corresponding author by a reasonable request.
